# Facile Solution Processing of Stable MXene Dispersions towards Conductive Composite Fibers

**DOI:** 10.1002/gch2.201900037

**Published:** 2019-07-15

**Authors:** Shayan Seyedin, Jizhen Zhang, Ken Aldren S. Usman, Si Qin, Alexey M. Glushenkov, Elliard Roswell S. Yanza, Robert T. Jones, Joselito M. Razal

**Affiliations:** ^1^ Institute for Frontier Materials Deakin University Geelong VIC 3216 Australia; ^2^ Research School of Chemistry The Australian National University Canberra ACT 2601 Australia; ^3^ Research School of Electrical, Energy and Materials Engineering The Australian National University Canberra ACT 2600 Australia; ^4^ Department of Chemistry and Physics Centre for Materials and Surface Science La Trobe University Bundoora VIC 3083 Australia

**Keywords:** composite fibers, MXenes, solution processing

## Abstract

2D transition metal carbides and nitrides called “MXene” are recent exciting additions to the 2D nanomaterials family. The high electrical conductivity, specific capacitance, and hydrophilic nature of MXenes rival many other 2D nanosheets and have made MXenes excellent candidates for diverse applications including energy storage, electromagnetic shielding, water purification, and photocatalysis. However, MXene nanosheets degrade relatively quickly in the presence of water and oxygen, imposing great processing challenges for various applications. Here, a facile solvent exchange (SE) processing route is introduced to produce nonoxidized and highly delaminated Ti_3_C_2_T*_x_* MXene dispersions. A wide range of organic solvents including methanol, ethanol, isopropanol, butanol, acetone, dimethylformamide, dimethyl sulfoxide, chloroform, dichloromethane, toluene, and *n*‐hexane is used. Compared to known processing approaches, the SE approach is straightforward, sonication‐free, and highly versatile as multiple solvent transfers can be carried out in sequence to yield MXene in a wide range of solvents. Conductive MXene polymer composite fibers are achieved by using MXene processed via the solvent exchange (SE) approach, while the traditional redispersion approach has proven ineffective for fiber processing. This study offers a new processing route for the development of novel MXene‐based architectures, devices, and applications.

## 1 Introduction

One of the paradigm shifts in 2D nanomaterial research is the increasing capacity to delaminate and exfoliate monolayer and few‐layer nanosheets from their parent bulk materials in large scale and high throughput.^1^, ^2^, ^3^, ^4^ Specifically, liquid phase exfoliation (LPE) techniques have been shown effective in maintaining the structural and chemical integrity of delaminated nanosheets.^1^, ^2^, ^4^ The ability to simultaneously exfoliate, delaminate, and stabilize nanosheets in a form of colloidal dispersion by LPE is proving to be a very powerful platform for enabling many fundamental and applied technological advances. Solution processable graphene and its derivatives, boron nitride nanosheets, phosphorenes, and transition metal dichalcogenides and oxides are some of the 2D nanosheets that have been developed into novel formulations and inks for a wide range of applications.^3^, ^5^, ^6^, ^7^, ^8^, ^9^, ^10^, ^11^

A recent exciting addition to the 2D family is “MXene” that is derived from its parent ternary carbide or carbonitride called MAX phase. “M,” “A,” and “X” denote for early transition metal, group 13–16 element, and carbon and/or nitrogen, respectively.^12^, ^13^, ^14^, ^15^, ^16^ MXene dispersions are typically prepared using a nonconventional exfoliation process following the etching–intercalation–delamination sequence in which the “A” layer is first etched from the MAX phase using hydrofluoric acid (HF) or LiF/HCl mixture (in situ HF etching) followed by solvent intercalation to aid the final step of sonication‐assisted delamination in water.^17^ The resulting product is an aqueous MXene colloidal dispersion that when processed into various macroscopic forms (e.g., films), displays metallic conductivity exceeding that of any nanosheet‐based material known to date.^12^, ^13^, ^14^, ^15^, ^16^ Combined with the ability to intercalate various ions and molecules and its hydrophilic surface, MXene finds such wide ranging applications as supercapacitors,^12^, ^14^, ^18^ metal‐ion capacitors,^19^, ^20^ electromagnetic interface shielding,^21^ water purification,^22^, ^23^ dyes and heavy metals adsorption, photocatalysis,^24^, ^25^ and functional composites.^18^, ^26^

One of the biggest challenges in assembling MXenes into suitable structures and devices is that, like other nanosheets (e.g., phosphorenes),^4^ MXenes, e.g., Ti_2_CT*_x_* and Ti_3_C_2_T*_x_* (T and *x* refer to surface termination groups and their numbers) are susceptible to undesirable oxidation when stored in water.^25^, ^27^, ^28^, ^29^, ^30^, ^31^, ^32^ This can lead to eventual degradation of their structural morphology from sheets into particles and their properties such as electrical conductivity and capacitance. Here, we present new insights into the stability of Ti_3_C_2_T*_x_* MXene in dispersion, not only in the context of their morphological and chemical fingerprint transformations in water, but also in terms of their stability and dispersibility in nonaqueous solvents. We also provide insights into how the unwanted oxidation can be circumvented without the need to dry MXene into powder, allowing for continuous and simple processing of delaminated MXene.

In this work we use Ti_3_C_2_T*_x_* as it is the most widely studied MXene. Ti_3_C_2_T*_x_* MXene oxidation and nanosheet degradation occur once stored in water.^27^ Hence, unless it is used immediately after the synthesis, it is necessary for the delaminated MXene nanosheets to be collected, dried, and stored in vacuum or in an inert atmosphere to avoid their oxidation. However, drying presents challenges when delaminated MXenes are to be used for further solution‐based processing. Similar to other nanosheets, redispersion of the dried powder in water does not lead to fully delaminated MXenes. In addition, further sonication is necessary, consequently decreasing the sheet size, leading to MXenes with lower electrical conductivities.^33^, ^34^ In this work, we have particularly chosen to study the highly conductive LiF/HCl‐produced Ti_3_C_2_T*_x_* MXene because redispersing the dried powder produced by this method is presently more challenging to achieve than the HF‐produced MXene.^35^

We show how MXene oxidation can be prevented using a simple solvent exchange processing method. The results obtained in this work are significant to the understanding and utilization of MXene's chemistry, which is critical for the subsequent processing of MXene into novel architectures and devices since present synthesis and processing methods for MXene begin from an aqueous‐based dispersion. As an example, we demonstrate the fabrication of Ti_3_C_2_T*_x_* MXene‐based polymer composite fibers by wet‐spinning, which was enabled by highly stable delaminated MXene dispersions in suitable solvents. The MXene processed using the solvent exchange route leads to conductive fibers, while the traditional redispersed MXene dispersion does not achieve fibers with measurable conductivities, clearly demonstrating the advantages of the solvent exchange approach. The processing approaches presented in this work are also expected to be applicable to other MXenes to achieve nonoxidized stable dispersions and for the fabrication of other composite structures and devices.

## 2 Results and Discussion

**Figure 1:**
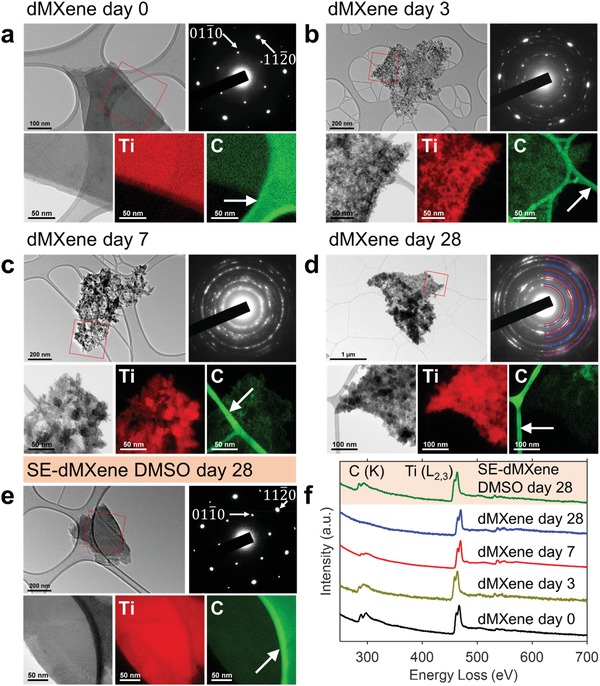
Structural and elemental identification of dMXene over time. a–e) Each frame contains TEM image, SAED pattern, energy‐filtered TEM (EFTEM) image of the selected area highlighted by a red box in the TEM image, elemental maps of the same for titanium and carbon. Red and blue overlays on the SAED in d) represent diffractions relating to TiO_2_ rutile and anatase phases respectively. Arrows on a–e) show the supporting carbon grid. f) Electron energy loss spectroscopy (EELS) results of dMXene sample over time and SE‐dMXene in dimethyl sulfoxide (DMSO) on day 28.

We synthesized Ti_3_C_2_T*_x_* MXene using the LiF/HCl approach.^14^ The details of MXene synthesis can be found in the Supporting Information, where we also provided full characterization data that validate the successful etching of aluminum layer, exfoliation of multilayer MXene (mlMXene) and delamination into single and few layer MXene (dMXene). The dark gray dMXene dispersion was free of large particles and contained monolayer and few‐layer flakes (**Figure** **1**a) with individual sheet thickness of ≈3 nm and lateral size of up to several micrometers (Figure S1 in the Supporting Information). The selected area electron diffraction (SAED) pattern of a representative dMXene flake obtained using transmission electron microscopy (TEM), showed a pattern of a single crystalline material, i.e., periodic arrays of spots (Figure 1a). A d‐spacing of ≈2.57 Å was measured from the (100) diffraction pattern. Electron energy loss spectroscopy (EELS) analysis showed prominent peaks at ≈284 and ≈465 eV related to carbon (C K‐edge) and titanium (Ti L_2,3_‐edge), respectively, and weak peaks at ≈540 to 550 eV indicating the minimal presence of oxygen (O K‐edge) as expected for freshly prepared dMXene (Figure 1f). The energy filtered TEM (EFTEM) elemental maps of Ti and C on a selected area of dMXene flake (Figure 1a) revealed that Ti and C were uniformly spread across the as‐synthesized (day 0) dMXene. The X‐ray diffraction (XRD) spectra of dMXene powder obtained by centrifugation and drying showed a sharp (002) diffraction at 2θ ≈ 6.3° and the disappearance of the prominent (104) diffraction of the MAX phase at 2θ ≈ 39° (Figure S1, Supporting Information), further confirming the complete etching and delamination.

Since we were interested in the behavior and characteristic of MXene in dispersions when stored for an extended period, we monitored the changes in dMXene dispersions and nanosheets over various storage times. Detailed investigation of time‐dependent oxidation of dMXene in water can be found in the Supporting Information, where we provided full characterization data of dMXene dispersions and flakes between 0 and 28 d after the synthesis. A commonly used indicator to identify the dMXene oxidation state is the change in the color of the dispersion. At ≈0.5 mg mL^−1^, the dark gray color of the dispersion turned lighter as early as 3 d and then became off‐white by day 14 and milky white by day 28 (Figure S4a, Supporting Information). The aqueous‐based oxidation of Ti_3_C_2_T*_x_* MXene (into TiO_2_) has been reported previously and is believed to be the result of dissolved oxygen in the dispersion.^24^, ^27^ Analysis of the SAED patterns (Figure 1d) identified the presence of TiO_2_ rutile and anatase phases in agreement with the XRD results (Figure S4d, Supporting Information). We further probed the dMXene nanosheet transformations by EFTEM mapping and EELS analyses. The EFTEM maps showed that by day 3, the representative flakes were comprised of Ti‐rich regions and C‐deficient regions (Figure 1b) unlike in day 0 where the Ti and C signals were homogeneously dispersed throughout the flake (Figure 1a). The signal for C decreased over time (Figure 1c) and was almost absent on day 28 (Figure 1d). Notably, we found that the decrease in C signal occurred randomly across the MXene flakes with almost no indication of preference to the edges, in contrast to recent reports for HF‐produced dMXene^27^ and for black phosphorous nanosheets^4^ where oxidation was observed to begin from the edges. Further confirming our results, EELS analyses of the representative flakes also showed a decrease in carbon K‐edge at ≈284 eV and a slight increase in oxygen K‐edge at 540 to 550 eV with prolonged storage (Figure 1f). Ti was found on all samples but only a small amount of C could be detected on day 28.

The oxidative degradation of dMXene can be temporarily prevented by collecting and drying them as powder or as film using centrifugation, filtration, and possibly by spray drying and freeze drying processes. However, the dried powders and films achieved by these approaches, are difficult to redisperse in water and in organic solvents without the need for prolonged sonication (3 h or more) particularly for the highly conductive LiF/HCl‐produced dMXene.^35^ Sonication results in sheet size reduction and small MXene nanosheets that have faster degradation and lower electrical conductivities than large sheets.^27^, ^33^ It is, therefore, ideal if dMXene can be redispersed using a sonication‐free process that simultaneously retains the delaminated state of the nanosheets.

**Figure 2:**
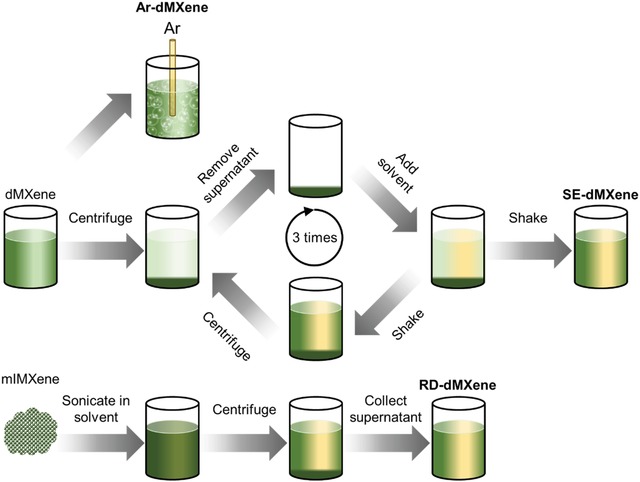
Schematic illustration of the three approaches used in this work to achieve stable dMXene dispersions in solution. These approaches include purging the dMXene water dispersion with argon (Ar‐dMXene), the solvent exchange processing approach (SE‐dMXene) developed in this work, and redispersion of mlMXene powder in organic solvents (RD‐dMXene).

We have investigated the stability and dispersibility of dMXene dispersions and nanosheet properties in various organic solvents using a very simple solvent exchange (SE) process. These dispersions, referred to as SE‐dMXene, were transferred across organic solvents by replacing the original solvent (water in the initial dispersion) with an organic solvent using centrifugation, redispersing the flakes only by vigorous shaking (by hand or by vortex mixer) and repeating the process for at least three times (**Figure** **2**). The majority of the solvent was extracted in each step resulting in a minimal water content in the final dispersion (measured as less than 0.1 vol%). For comparison, we also studied dMXene dispersions prepared by the following two methods: 1) continuous purging of the aqueous dMXene dispersion with argon for the duration of the study (Ar‐dMXene) and 2) redispersion by direct delamination of the dried mlMXene powder in selected solvents (RD‐dMXene). The RD‐dMXene method was adopted based on the previous work by Maleski et al.^35^ This method required sonication for 1 h in the desired solvent followed by centrifugation to yield homogenous dMXene dispersions (Figure 2). All samples were kept in screw‐capped clear glass vials and placed on laboratory bench under ambient conditions.

**Figure 3:**
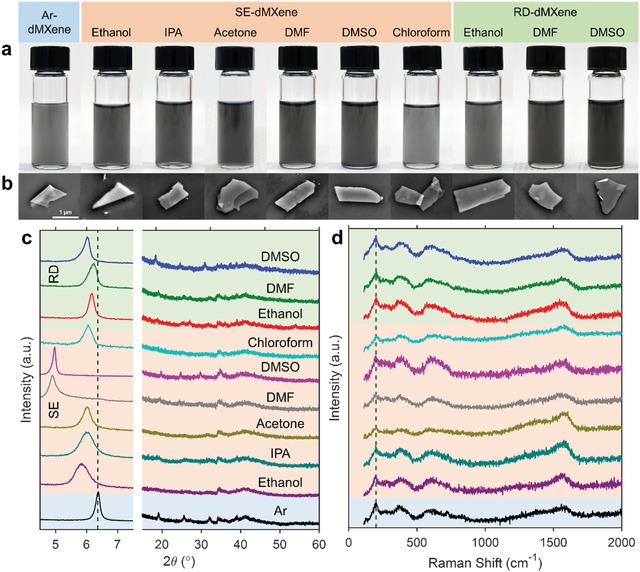
Characterizations of Ar‐, SE‐, and RD‐dMXene dispersions after storage for 28 d. a) Digital photographs and b) SEM images of the dispersions in vials 28 d after processing. c) XRD patterns of Ar‐, SE‐, and RD‐dMXene powders collected from their dispersions on day 28. The dashed line in (c) represents the (002) diffraction of the freshly prepared dMXene. d) Raman spectra of the Ar‐, SE‐, and RD‐dMXene powders on day 28. The color coding and positioning of the samples within the figure in (c) are the same as the samples in (d).

Transferring the dMXene dispersion into other solvents was straightforward using the SE process. SE‐dMXene was easily dispersed in polar solvents, e.g., methanol, ethanol, isopropanol (IPA), acetone, dimethylformamide (DMF), and dimethyl sulfoxide (DMSO). The dispersion quality was similar to the original dMXene dispersion in water without the need for sonication, unlike for RD‐dMXenes that required sonication (see the Supporting Information for detailed characterization of dMXene produced using different processing routes). Visual inspections of the SE‐dMXene dispersions showed that all dispersions remained dark (black or gray depending on the concentration) after 28 d suggesting that the oxidation did not occur (**Figure** **3**a). It was also possible to prepare sonication‐free nonoxidized dMXene dispersions in solvents previously shown poor or fair for LiF/HCl‐produced MXenes, even after prolonged sonication (i.e., methanol and acetone).^35^ When transferring to nonpolar solvents or to water immiscible solvents (e.g., butanol, chloroform, dichloromethane, toluene, and *n*‐hexane), a cosolvent (e.g., ethanol or acetone) in the first redispersion step of the SE process was used until miscibility was achieved. Most SE‐dMXene dispersions (except those in toluene and *n*‐hexane) remained free of large aggregates, unlike the Ar‐dMXene dispersions which aggregated over time observed through dynamic light scattering (DLS) measurements (Figure S9, Supporting Information). On the other hand, the RD approach only worked for a limited number of solvents (ethanol, DMF, and DMSO) for the MXene synthesized by the LiF/HCl approach while it was previously shown to achieve high‐quality dispersions in a wide range of solvents^35^ using MXene synthesized by the HF route. The use of other solvents in the RD approach resulted in very low yields (Figure S6, Supporting Information). The shape of the UV–vis absorbance spectra of all three dispersion types (SE and RD) remained the same until day 28 suggesting that MXene did not change in composition (Figure S8, Supporting Information).

Scanning electron microscopy (SEM, Figure 3b) and TEM (Figure 1e) studies revealed that the sheet morphology of all SE‐, Ar‐, and RD‐dMXene samples remained intact, with no evidence of particle nucleation even after 28 d. As an example, the SE‐dMXene sample in DMSO on day 28 (Figure 1e) and the dMXene on day 0 (Figure 1a) showed similar SAED patterns displaying the (100) and (210) diffractions with respective *d*‐spacings for the (100) diffraction of ≈2.62 and ≈2.57 Å. Furthermore, unlike oxidized dMXene in aqueous dispersions where the C signal in EELS was no longer detectable by day 28, the C signal for the SE‐dMXene sample on day 28 was similar to the dMXene on day 0 (Figure 1f). It was also apparent in the EFTEM maps that both Ti and C were present across the flake with no evidence of degradation in the C layer (Figure 1e).

XRD analyses corroborate well with the above findings (Figure 3c). The XRD diffraction peaks for dMXene, i.e., (002), were present in all powder samples collected from SE‐, Ar‐, and RD‐dMXene dispersions. Notably, there was no evidence of diffraction patterns for TiO_2_ even for those collected and dried on day 28. Raman spectroscopy also confirmed the absence of TiO_2_ on all samples by day 28 (Figure 3d). The significant differences between the samples were observed in the XRD patterns on the extent of downshifts in the (002) diffraction positions (Figure 3c). These downshifts (increased *d*‐spacing) have been previously attributed to the efficacy of solvent intercalation during delamination, particularly for HF‐produced MXenes.^12^, ^13^, ^36^ In our case, the (002) diffraction peak for SE‐dMXene in DMSO at 2θ ≈ 5.1°, which corresponded to a *d*‐spacing of 17.2 Å, is higher than that of the parent (aqueous) dMXene sample (13.9 Å). Notably, the (002) diffraction positions of all SE‐dMXene downshifted relative to the parent dMXene. For the SE‐dMXene in DMSO, the presence of the intercalated solvent was also seen in the X‐ray photoelectron spectroscopy (XPS) spectra manifested by the S 2s and S 2p peaks (Figure S12, Supporting Information). When we investigated the (002) diffraction in the XRD spectra of SE‐dMXene powders collected at different time points, further downshifts were observed over time for some solvents (e.g., ethanol, IPA, and DMF). In DMF for instance, the (002) diffraction shifted from 2θ ≈ 6.0° (*d*‐spacing ≈ 14.8 Å) to ≈4.9° (*d*‐spacing ≈18.0 Å). The RD‐dMXene samples, which were dried prior to redispersion in solvents showed lower *d*‐spacings compared to the SE‐dMXene samples. It could be inferred from these results that the SE process allowed for further delamination of the MXene sheets because solvent intercalation became more effective when dMXenes were not fully dried.

This oxidation of dMXenes in aqueous media can be prevented by eliminating one of the two oxidizing components (water or oxygen). This can be accomplished either by de‐aerating the dispersion with argon (removing oxygen) or by using organic solvents to disperse and store dMXene (removing water). Our results suggest that the activity of dissolved oxygen in water favors the oxidation of dMXene over that of oxygen in organic solvents. The difference in reduction potentials when dMXenes are in different oxidizing environments require further detailed study. Nevertheless, the use of organic solvents whether by solvent exchange (SE) or by redispersion (RD) afford effective routes for liquid‐based processing of nonoxidized highly delaminated MXene. The SE route allows for increased delamination as shown by increased *d*‐spacing from solvent intercalation, which could be beneficial for applications requiring intercalation of various species such as in supercapacitors or composites. The SE route can also preserve the sheet size of MXene. This might be particularly advantageous when high conductivity is required. On the other hand, the RD route requires sonication that can result in low MXene sheet size, works with only a limited number of solvents for LiF/HCl‐derived MXene, and typically achieves lower delamination compared to the SE route.

We further demonstrated the versatility of the SE approach by sequentially transferring the dMXene dispersions to another solvent using the SE route for a number of times (*n*). The resulting dispersions were referred to as *n*SE‐dMXene. As an example, we have carried out the SE process for up to five times (Figure S7a, Supporting Information) and sequentially transferred the SE‐dMXene methanol dispersion (transferred initially from water via the SE route) to DMSO (2SE), chloroform (3SE), ethanol (4SE), and acetone (5SE). This 5SE‐dMXene dispersion showed no evidence of aggregation or oxidation for 28 d. The SE‐dMXenes can also be returned to water (Figure S7b, Supporting Information). This means that the dMXene flakes can be stored first in an organic solvent for an extended period to prevent the oxidative degradation while maintaining their delaminated state and then transferred back to water only when necessary (to meet the requirements of the solution processing to be used). It is also possible to begin with either the RD route or any of the recently published methods and then use the SE processes to realize dispersions in solvents not feasible using those approaches. These alternative routes may be preferred if using dried mlMXene or dMXene powder as starting materials although sonication may be required for the initial step therefore an all‐SE route seem more practical.

**Figure 4:**
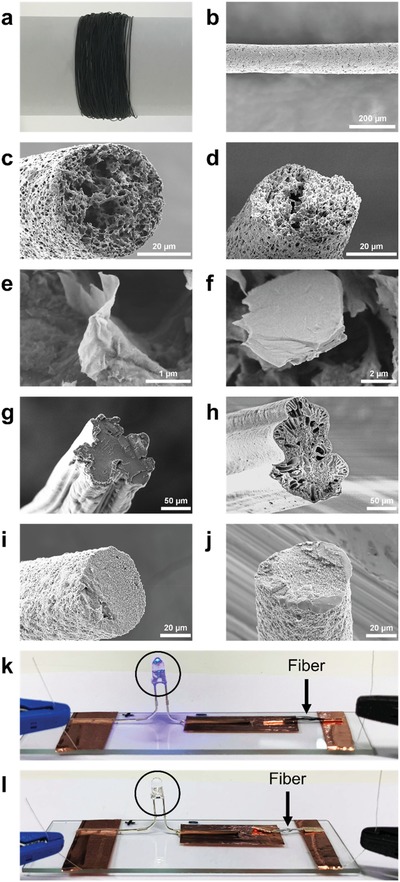
dMXene composite fibers produced by wet‐spinning. a) Digital photograph showing wet‐spun SE‐dMXene/PCL fiber with MXene to PCL ratio of 3:10. SEM images showing b) surface morphology of the SE‐dMXene/PCL fiber and cross‐section morphologies of c) SE‐dMXene/PCL and d) RD‐dMXene/PCL fibers; e,f) Higher magnification SEM images of the fibers in (c) and (d), respectively. SEM images of cross‐section morphologies of g) SE‐dMXene/PAN, h) RD‐dMXene/PAN, i) SE‐dMXene/PVDF, and j) RD‐dMXene/PVDF composite fibers. k) SE‐dMXene/PCL and l) RD‐dMXene/PCL fibers were connected in series to a blue light‐emitting diode (LED). Arrows show the position of the fibers. Only the SE‐dMXene/PCL fiber could turn on the LED.

The solvent processing of dMXene enabled the integration of MXene nanosheets within various polymer matrices, which often use organic solvent media. We used the wet‐spinning approach^37^, ^38^, ^39^, ^40^, ^41^, ^42^, ^43^, ^44^, ^45^, ^46^ and successfully produced MXene composite fibers with a number of polymers, e.g., polycaprolactone (PCL, **Figure** **4**a–f), polyacrylonitrile (PAN, Figure 4g,h) and polyvinylidene fluoride (PVDF, Figure 4i,j). The MXene dispersions processed via solvent exchange (SE‐dMXene) and redispersion (RD‐dMXene) routes were first mixed with the polymer with different ratios (i.e., 1:10, 2:10, and 3:10) in DMF and then spun into fibers using IPA as the coagulating solvent. While it was possible to achieve fibers continuously (Figure 4a) using both MXene dispersions, the SE‐dMXene resulted in smoother and more homogeneous fibers with better distribution of MXene nanosheets within the polymer host as seen in high magnification SEM images for the PCL composite fibers (Figure 4e,f). More importantly, we found that the SE‐dMXene/PCL fiber became conductive when the MXene/PCL ratio reached 3:10 (MXene loading ≈23 wt%), while the RD‐dMXene/PCL fiber remained insulating. This result suggests that dMXene processed using the SE route promotes the formation of a continuous percolation network in the polymeric host, which is likely due to the larger sheet size and less stacking of the dMXene prepared using this approach. We tested ≈0.5 cm long SE‐dMXene/PCL fiber with the MXene loading of ≈23 wt% and measured a resistance of ≈2.14 MΩ, equivalent to an electrical conductivity of 1.84 mS cm^−1^. This conductivity is higher than that of previous MXene composite films. For instance, Ti_3_C_2_T*_x_*/polyvinyl alcohol (PVA) composite film showed a conductivity of ≈0.4 mS cm^−1^ at even a high MXene loading of 40 wt%.^26^ When used as the conductive wire to complete a circuit, the SE‐dMXene/PCL fiber was able to turn on a light emitting diode (LED) as shown in Figure 4k. This demonstration clearly shows that the solvent exchange route is an effective method for processing highly delaminated MXene dispersions, which could be easily integrated with various conventional processes for building functional MXene architectures. While the addition of dMXene resulted in a slight decrease in the tensile strength and the strain at break of the pristine polymer fibers, the SE‐dMXene led to lower overall mechanical properties deterioration. For instance, the SE‐dMXene/PCL fiber showed a higher stretchability than the RD‐dMXene/PCL fiber (770% vs 622%) at the same MXene content (MXene/PCL ratio of 3:10). Table S3 (Supporting Information) summarizes the mechanical properties of MXene/PCL composite fibers.

## 3 Conclusions

In this work, we presented a versatile solvent exchange (SE) approach to achieve stable, nonoxidized, and aggregate‐free dMXene dispersions in various organic solvents. The SE approach does not require sonication, is efficient and effective in delaminating MXene, and can be carried out multiple times in sequence to transfer dMXene across various solvents and back to water if and when desired. The SE approach provides outstanding versatility to storage, handling and processing of dMXene. We showed that the SE approach introduced in this work could utilize the electrical properties of Ti_3_C_2_T*_x_* MXene in polymer composite systems over the conventional method of redispersing MXene powders in organic solvents. We used dMXene prepared by the SE route and fabricated conductive MXene polymer composite fibers. This work demonstrated a feasible approach to change solvents on demand from a wide range of solvents with very little or no loss, offering a versatile route to solvent‐based processing of novel MXene‐based macroscopic assemblies and devices.

## 4 Experimental Section

*Transferring dMXene into Organic Solvents by Solvent Exchange without Drying and Sonication*: Aqueous dMXene dispersions were transferred into selected organic solvents, i.e., methanol, ethanol, isopropanol (IPA), butanol, acetone, dimethylformamide (DMF), dimethyl sulfoxide (DMSO), chloroform, dichloromethane (DCM), toluene, and *n*‐hexane by using a simple solvent exchange (SE) method. Briefly, this method involved repeated centrifugation (ThermoScientific Heraeus Multifuge X3R) of the parent aqueous dMXene dispersion at 15 000 rpm (24 630 g) for 1 h. In each centrifugation step, the supernatant was carefully removed and replaced with the chosen solvent and redispersion was achieved by shaking the dispersion for 5 min using a vortex mixer. This process was repeated for at least three times to remove as much water as possible. These dispersions were referred to as “SE‐dMXene.” The same procedure was followed for the sequential solvent transfer experiments. Detailed experimental procedures including materials used, synthesis of multilayered MXene (mlMXene), its delamination in water (dMXene), and redispersion in organic solvents (RD‐dMXene) can be found in the Supporting Information.

*Wet‐Spinning of MXene Polymer Composite Fibers*: The Ti_3_C_2_T*_x_* MXene composite fibers were prepared using a wet‐spinning^37^, ^38^, ^39^, ^40^, ^41^, ^42^, ^43^, ^44^, ^45^, ^46^ approach. First, Ti_3_C_2_T*_x_* MXene dispersions in DMF (concentration of 30 mg mL^−1^) were achieved using SE and RD approaches. The spinning solution (spinning dope) was then prepared by adding various polymers, i.e., polycaprolactone (PCL, MW = 80 kDa), polyacrylonitrile (PAN), or polyvinylidene fluoride (PVDF), to achieve MXene to polymer ratios of 1:10 to 3:10. The mixture was stirred for 8 h at 60 °C until the polymer was dissolved and homogeneous formulation was obtained. The wet‐spinning was carried out by loading the spinning dope into a 1 mL syringe and continuously injecting the formulations into a coagulation bath of IPA through a needle (25 gauge, inner diameter 0.26 mm) at a flow rate of 1.5 mL h^−1^. The resulting fiber was then dried and collected on a spool. The collected fibers were stored in vacuum until used for further investigation.

*Characterizations*: Scanning electron microscopy (SEM) images of the samples were taken using a field emission SEM (JEOL JSM‐7800F). MAX phase and mlMXene samples were prepared by spreading a small amount of powder on a carbon tape attached to an aluminum specimen stub. dMXene samples from dispersions were deposited onto salinized silicon wafers (SiO_2_ layer thickness 285 nm) by dipping the wafers into a diluted dMXene dispersion (≈50 µg mL^−1^) and air‐drying. Silicon wafers were salinized by immersing into a solution of 3‐aminopropyltriethoxysilane in water (1:9 v/v) to which a drop of HCl was added. Transmission electron microscopy (TEM) studies were carried out using a JEOL JEM‐2100F instrument. An accelerating voltage of 200 kV was used to observe the MXene flakes. The TEM samples were prepared by dipping a 300‐mesh holey carbon‐coated copper grid into a diluted dispersion of MXene (≈50 µg mL^−1^) and subsequent air‐drying. Electron energy loss spectroscopy (EELS) and energy filtered TEM (EFTEM) analyses were carried out using a Gatan Quantum ER 965 Imaging Filter installed on the JEOL JEM‐2100F microscope. EELS spectra were acquired in the image‐coupled mode from the areas defined by the SAED aperture. Three‐window method was used for acquiring energy‐filtered elemental maps.

X‐ray diffraction (XRD) patterns were recorded with a powder diffractometer (PANalytical X'Pert Powder) using Cu Kα radiation (λ = 1.54 Å) at a 2θ scan step of 0.013° and 100 s dwell time. XRD samples were prepared by spreading the powder on a zero XRD diffraction SiO_2_ substrate.

Raman spectroscopy was performed using Renishaw InVia Raman Microspectrometer equipped with an Ar^+^ ion laser (514 nm excitation wavelength, 50 mW). The spectra of dMXene powders were recorded over 100 to 2000 cm^−1^ range using a 50× objective (0.42 µm spot), a 2400 line mm^−1^ grating, 50 seconds accumulation time, and 5% laser power.

The morphology of the Ti_3_C_2_T*_x_* MXene composite fibers was observed using an SEM. The diameter of the fibers was measured from SEM images on at least ten positions along their length. Tensile testing was carried out using an Instron 30 kN Universal Testing Machine with a load cell of 5 N. The composite fibers were cut into 20 mm long snippets and were taped onto supporting paper frames with gauge length of 10 mm. Tensile tests were performed at a strain rate of 100% min^−1^. The tensile strength and strain at break values were calculated from the stress‐strain curves for at least five samples. The electrical conductivity of the composite fibers was measured using a digital multimeter (Keysight 34461A) on an in‐house, linear four‐point probe cell under laboratory humidity and temperature conditions.

## Conflict of Interest

The authors declare no conflict of interest.

## Supporting information

Supplementary
