# Supplementary material for: Facile Solution Processing of Stable MXene Dispersions towards Conductive Composite Fibers
Source: Glob Chall. 2019 Jul 15;3(10):1900037. doi: 10.1002/gch2.201900037 (PMC6777206; doi:10.1002/gch2.201900037)
Supplement: Supplementary file 1 — Supplementary [file GCH2-3-1900037-s001.pdf]

Copyright WILEY-VCH Verlag GmbH & Co. KGaA, 69469 Weinheim, Germany, 2019.

Global Challenges
Open Access

Supporting Information

for *Global Challenges,* DOI: 10.1002/gch2.201900037

Facile Solution Processing of Stable MXene Dispersions towards Conductive Composite Fibers

Shayan Seyedin, Jizhen Zhang, Ken Aldren S. Usman, Si Qin, Alexey M. Glushenkov, Elliard Roswell S. Yanza, Robert T. Jones, and Joselito M. Razal**

Copyright WILEY-VCH Verlag GmbH & Co. KGaA, 69469 Weinheim, Germany, 2019.

Supporting Information

Facile Solution Processing of Stable MXene Dispersions towards Conductive Composite Fibers

Shayan Seyedin, Jizhen Zhang, Ken Aldren S. Usman, Si Qin, Alexey M. Glushenkov, Elliard Roswell S. Yanza, Robert T. Jones and Joselito M. Razal**

Dr. S. Seyedin, J. Zhang, K.A.S. Usman, Dr. S. Qin, Dr. A.M. Glushenkov, E.R.S. Yanza, and Prof. J.M. Razal
Deakin University, Institute for Frontier Materials, Geelong, VIC 3216, Australia
E-mail: joselito.razal@deakin.edu.au, shayan.seyedin@deakin.edu.au

Dr. A.M. Glushenkov
The Australian National University, Research School of Electrical, Energy and Materials Engineering, Canberra, Australia

Dr. R.T. Jones
La Trobe University, Centre for Materials and Surface Science, Department of Chemistry and Physics, Bundoora, VIC 3083, Australia

1. Supplementary experimental

1.1. Materials

Ti_3AlC_2 powder (MAX phase) with particle size < 40 µm was purchased from Carbon Ukraine. Methanol, ethanol, isopropanol (IPA), butanol, acetone, dimethylformamide (DMF), dimethyl sulfoxide (DMSO), dichloromethane (DCM), chloroform, toluene, and *n*-hexane were purchased from Chem-Supply and used without further purification. Lithium fluoride (LiF, 98+%, Bio-Scientific), hydrochloric acid (HCl, 37%, Sigma-Aldrich), and 3-aminopropyltriethoxysilane (Sigma–Aldrich) were used as received.

1.2. Synthesis of multi-layered MXene

$Ti_3C_2T_x$ MXene was synthesized using the LiF/HCl method.[1] In a typical synthesis, LiF (1.98 g) was dissolved in HCl (6 $_M$, 30 mL) in a PTFE beaker. Ti_3AlC_2 MAX phase powder (3 g) was slowly added to the solution. The mixture was heated to 40 °C and stirred for 45 h in a sealed beaker. The mixture was then diluted with water (Milli-Q) for up to 50 times and then centrifuged (Beckman J2-MC) at 7,500 rpm (8,630 g) for 15 minutes. Washing with centrifugation was repeated at least five times until the supernatant reached above *pH* ~6. The dispersion obtained at this stage contained multi-layer MXene nanosheets and referred to as "mlMXene".

1.3. Delamination of mlMXene in water

To obtain few- and/or single-layer MXene flakes, the washed ml-MXene dispersion was immediately bath-sonicated (Unisonics FXP12M, 100 W) for 1 h under continuous bubbling of argon. The sonicated dispersion was then centrifuged (Beckman J2-MC) at 500 rpm (38 g) for 1 h and the supernatant containing delaminated MXene was collected (concentration ~0.5 mg mL^{-1}) and referred to as "dMXene".

1.4. Drying of mlMXenes and re-dispersion in organic solvents

The aqueous mlMXene dispersion was filtered using a 0.22 µm PTFE filter membrane, dried and stored in a desiccator under vacuum until ready for use. This mlMXene powder was re-dispersed and delaminated in organic solvents (ethanol, IPA, acetone, DMF, DMSO, chloroform and toluene) by adding the mlMXene powder (2 g) into the solvent (400 mL). The mixture was stirred for 18 h at room temperature prior to bath sonication for 1 hr. The resulting dispersion was centrifuged at 500 rpm (38 g) for 1 h (Beckman J2-MC) and the supernatant containing the delaminated MXene was collected. These dispersions were referred to as “RD-dMXene”.

1.5. Atomic force microscopy of dMXene

The atomic force microscopy (AFM) images of dMXene samples were obtained using Multimode 8-U AFM (Bruker) in a tapping mode (ScanAsyst) using a SCANASYST-AIR probe (resonant frequency 70 kHz and spring constant 0.4 N m^{-1}). Samples were prepared by drop-casting a very dilute dMXene dispersion (~50 µg mL^{-1}) on a mica substrate and then air-dried.

1.6. Characterizations

UV-Vis absorbance spectra of the dMXene dispersions were acquired on Cary 3 UV-Visible Spectrophotometer (Varian) using quartz cuvettes with 10 mm path length (Starna Pty. Ltd.). The average size and ζ-potential of dMXene in dispersions were measured by Zetasizer (Malvern Nano-ZS) using disposable folded capillary cells (Malvern DTS 1070). Each test was repeated for at least three times and the dispersions were vigorously shaken before the measurements.

X-ray photoelectron spectroscopy (XPS) data were acquired on a Kratos AXIS Nova instrument (Kratos Analytical Ltd) equipped with a monochromated Al Kα source ($h\nu$ = 1486.6 eV) operating at 150 W. The samples were immobilized for the analysis by pressing

them onto a double-sided carbon tape. Survey spectra were acquired at a pass energy of 160 eV and at an energy interval of 1 eV. The Ti 2p, C 1s, and O 1s spectra were acquired at a pass energy of 20 eV and an energy interval of 0.1 eV. The data were processed using the CasaXPS software program (Casa Software Ltd).

2. Supplementary results and discussion

2.1. Validation of mlMXene synthesis

$Ti_3C_2T_x$ MXene synthesis was carried out using previously established LiF/HCl method[1] as described in detail in the Supplementary experimental section. Here we first validate the successful etching of the aluminum layer from MAX phase by scanning electron microscopy (SEM), X-ray diffraction (XRD), and XPS analyses. SEM images (**Figure S1a,b**) showed that the MAX phase was carved into separated sheets after etching suggesting the successful production of multi-layer MXene (mlMXene). This observation was confirmed by XPS and XRD results. XPS revealed that the characteristic Al 2p peak seen in MAX phase was absent in mlMXene (**Figure S2**). X-ray diffraction (XRD) analysis showed that the prominent (104) diffraction pattern at 2θ ~ 39° for aluminum seen in MAX phase was no longer visible in mlMXene (**Figure S1e**). Furthermore, all (00*l*) peaks shifted to lower 2θ after etching and all except the (002) diffraction pattern broadened and their intensities decreased. This (002) peak for mlMXene increased in intensity and sharpness, and shifted to 2θ ~6.5° from ~9.5° (for MAX phase). Based on this (002) diffraction, the interlayer spacing increased to 13.5 Å in mlMXene from 9.3 Å in MAX phase. These results are in accordance with literature reports.[1–5]

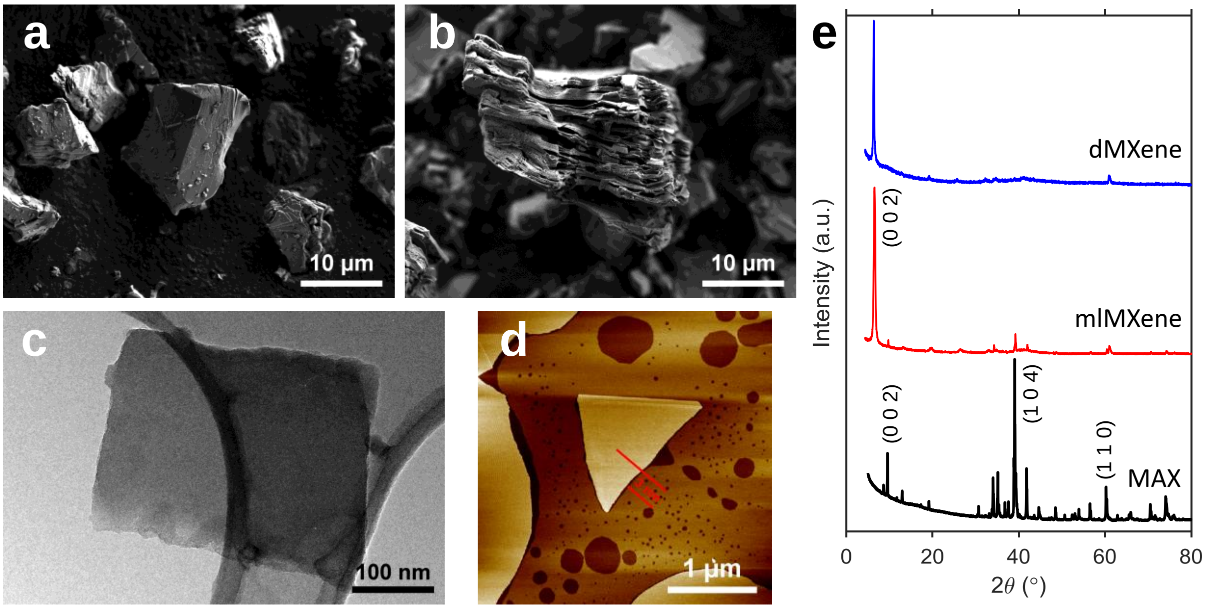

Figure S1. Evidence of dMXene synthesis. SEM images of (a) Ti_3AlC_2 (MAX phase) and (b) $Ti_3C_2T_x$ (MXene) obtained through the LiF/HCl etching process. (c) TEM and (d) AFM images of a dMXene flake. The lateral dMXene sheet size exceeds 1 µm. The AFM image shows a layer thickness of ~3 nm. (e) XRD spectra of MXene at different stages of synthesis. The (104) diffraction of MAX phase disappeared after etching and the (002) diffraction downshifted and gained intensity providing evidence for removal of Al and delamination of MXene respectively.

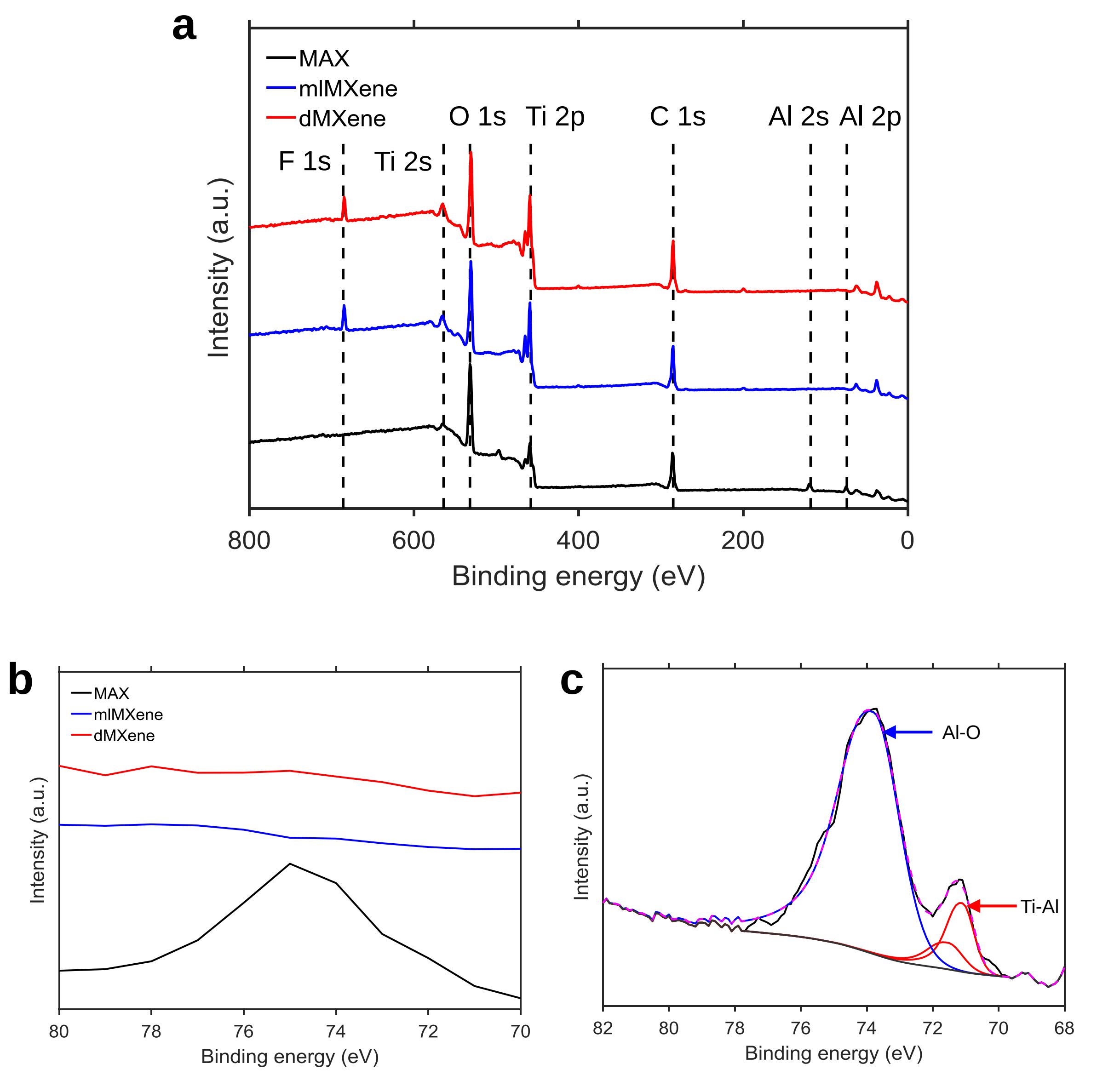

Figure S2. XPS spectra of MAX, mlMXene, and dMXene powders. Survey spectra for (a) entire binding energy range and (b) between 70-80 eV showing the presence of Al 2p peak for MAX and its absence for mlMXene and dMXene indicating the removal of Al after etching. (c) High resolution XPS spectrum of MAX phase for Al 2p region indicating the presence of the Ti-Al bond that is absent in mlMXene and dMXene (b).

2.2. Oxidation study of dMXene in water

Relevant reports on $Ti_3C_2T_x$ MXene stability have proven that MXene oxidation can proceed under several conditions. Studies involving flash heating,[6,7] hydrothermal,[7,8] and solvothermal[9] treatments of multi-layered MXene revealed the formation of TiO_2 particles on nanosheet edges, which were found to be predominantly in the anatase phase. Similar conclusions were reached when $Ti_3C_2T_x$ MXene was stored in water (in the presence of oxygen).[10] An earlier report had indicated the presence of both rutile and anatase phases upon storage of MXene in water.[11] Contrasting these reports which were carried out for HF-derived MXenes, here we show that $Ti_3C_2T_x$ MXenes obtained by the LiF/HCl method degrade primarily as rutile TiO_2 and this degradation occurs randomly on the sheet surface (**Figure S3**).

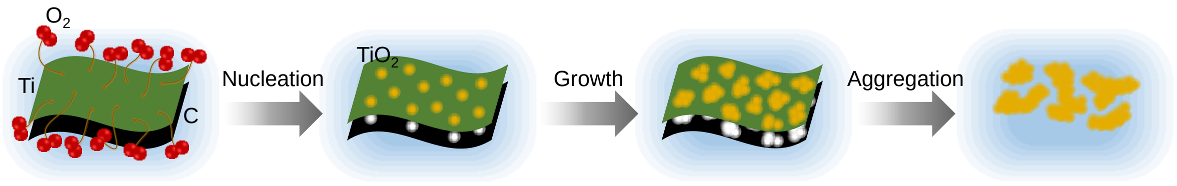

Figure S3. Schematic illustration of the proposed stages of MXene oxidation in water. The oxidation of MXene begins with nucleation of TiO_2 particles and their growth over time to form clusters that then coalesce to form large aggregates.

A gradual decrease in the UV absorbance was observed with increasing the storage time (**Figure S4c**). Also, the wavelength at maximum absorbance (λ_{max}) shifted from ~278 nm (day 0) to ~368 nm (day 28) and the broad peak between 700 and 800 nm gradually disappeared. This result agrees well with and follows the trend reported in the previous work on the oxidation of the $Ti_3C_2T_x$ MXene synthesized by HF route.[10] Our electron microscopy studies suggested that the dMXene oxidation process began with TiO_2 nucleation, which grew over time to form TiO_2 clusters and finally coalesced to form large TiO_2 aggregates. This

morphological transformation from sheets to aggregated particles with prolonged storage, shown schematically in **Figure S3**, was clearly evident from the SEM images shown in **Figure S4b**.

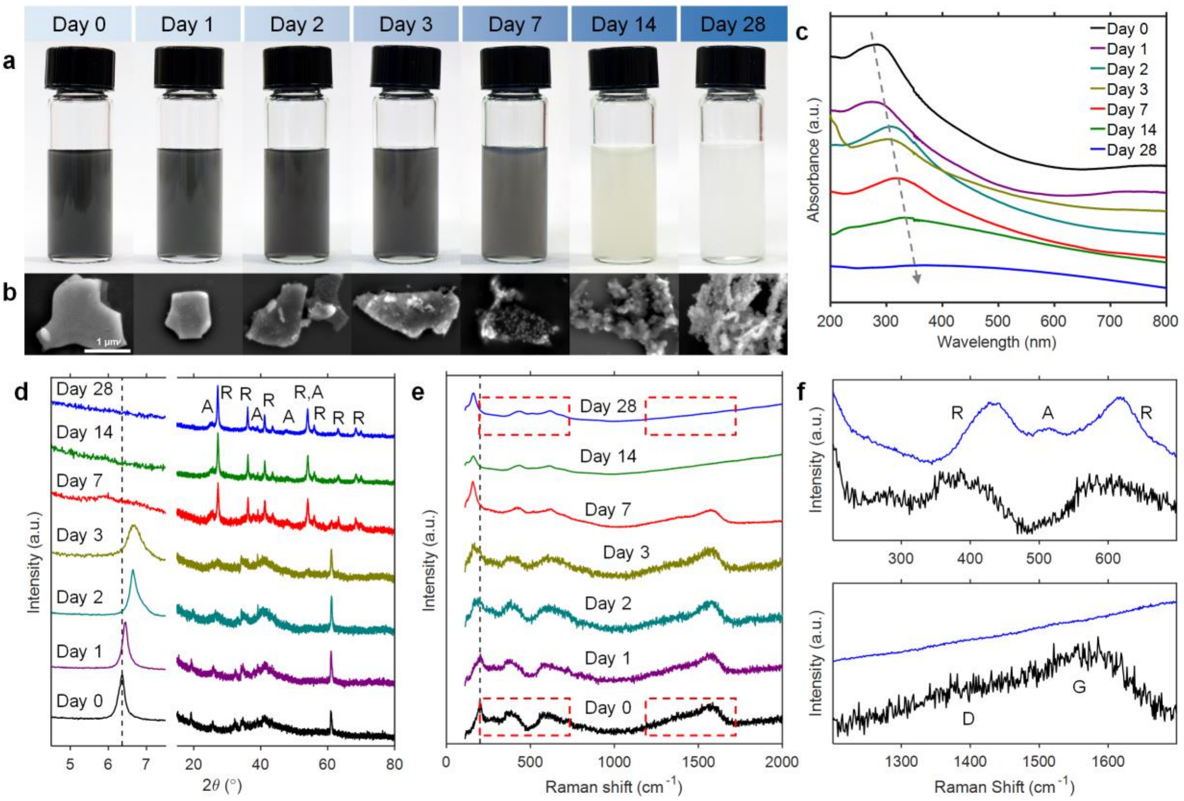

Figure S4. Oxidation of dMXene in water. (a) Digital photographs and (b) SEM images of the dMXene dispersion in a vial 0-28 days after the synthesis. (c) UV-Vis absorbance spectra of the dMXene dispersions over time. (d) XRD patterns of the dMXene powders collected at different storage periods. The dashed line in (d) represents the (002) diffraction of the freshly prepared dMXene. Diffractions relating to TiO_2 rutile and anatase phases are marked by (R) and (A) respectively in (d). (e) Raman spectra of the dMXene powders from day 0 to day 28. (f) Superimposed Raman spectra of days 0 and 28 for the 1200-1700 cm^{-1} and 200-700 cm^{-1} regions highlighted by red boxes in (e). D and G bands vibrations of the carbon layer of MXene are as marked in (f). Vibrations relating to TiO_2 rutile and anatase phases are represented in (f) by (R) and (A) labels respectively.

We also investigated the oxidization of dMXene by XRD, Raman analyses and XPS of the dried powder collected from each dispersion. The XRD spectra show that the (002) diffraction of dMXene broadened and upshifted by day 3, decreased in intensity by day 7, and disappeared by day 14 (**Figure S4d**). These results indicated the loss of crystallinity and structural order of dMXene, and its transformation into other compositions. Concomitantly, new diffraction peaks emerged on day 7, which matched the TiO_2 rutile (JCPDS No. 21-1276) and TiO_2 anatase (JCPDS No. 21-1272) phases. When the samples were assumed to only comprise of these two TiO_2 phases, quantitative analysis of the XRD data revealed a TiO_2 composition of ~87 % rutile and ~13 % anatase phases, which were surprisingly found to remain constant throughout the oxidation period. Raman spectra (**Figure S4e**,**f**) showed that the Raman signal attributed to carbon vibrations, *i.e.* G band at ~1570 cm^{-1} and D band at ~1360 cm^{-1}, disappeared on day 14. The out-of-plane stretching (A_{1g}) vibration of Ti in MXene[12] at ~200 cm^{-1} broadened on day 3. On day 7, a new sharp peak at ~160 cm^{-1} appeared, which could be attributed to the doubly degenerate (E_g) vibrations of Ti in TiO_2 anatase phase.[13,14] On day 28, the broad Raman signals for pristine (day 0) dMXene at ~373 cm^{-1} and ~581 cm^{-1} shifted to ~433 cm^{-1} and ~617 cm^{-1}, respectively. These signals could be attributed to the E_g and in-plane stretching (A_{1g}) vibrations of oxygen in TiO_2 rutile phase, respectively.[6,7,13,14] Also, a broad peak emerged at ~512 cm^{-1}, which could be assigned to the antisymmetric (A_{1g}) bending vibrations of oxygen in TiO_2 anatase phase.[6,7,13,14]

This is in agreement with theoretical studies where MXene surfaces in aqueous medium are shown to be saturated with oxygen that can dissociate and readily diffuse into MXene layers.[15,16] From these studies, it can be deduced that the oxidation process begins with oxygen diffusing into the MXene lattice, resembling the oxidation of titanium carbide (TiC) powder in water.[17] The dissolved oxygen can form strong covalent bonds with the Ti and C atoms of MXene.[15] However, oxidation of Ti occurs first before C because of the more negative Gibbs free energy of Ti oxidation than C.[18] This aqueous-based oxidation process is

slower than the oxidation of MXene in air by flash heating,[6] therefore we can expect that over time, the oxidation of C also takes place in regions where Ti oxidation has already occurred to form TiO_2-rich regions and C-deficient regions on MXene surfaces as illustrated in **Figure S3**.

We also measured the ζ-potential and *pH* of the dMXene aqueous dispersions over the storage period. We noticed that the fresh dMXene dispersion was slightly acidic with *pH* ~5, although the supernatant of this dispersion after centrifugation was relatively neutral and had a *pH* of ~6 (**Figure S5a**). For the freshly prepared dMXene dispersion, we found that the ζ-potential was -32 mV (at *pH* ~5), which further decreased to -35 mV when neutralized to *pH* ~7 by titrating with NaOH (**Figure S5b**). These results are comparable with values reported by Ying *et al.*[19] where ζ-potential in the range of +5 to -20 mV in acidic conditions and around -20 to -40 mV in neutral *pH* (6 to 8) were reported. Alhabeb *et al.*[20] reported more negative ζ-potential (below -30 mV at *pH* 4 to 8 and -60 in a basic conditions). This might be due to the different MAX phase (Ti_3SiC_2) used as the starting material. Monitoring the dMXene dispersion during the degradation process showed a slight decrease in the *pH* from ~5 to ~3.6 after 28 days (**Figure S5a**). Similarly the *pH* of the supernatant of the dMXene dispersion decreased from ~5.7 to ~3.6. We observed that the ζ-potential of the dMXene dispersion increased rapidly from -32 mV (day 0) to around -5 to -10 mV after 3 days (**Figure S5b**). Nevertheless, when the *pH* of the aliquot was adjusted to around 7 (by titrating with NaOH), the ζ-potential remained stable at around -30 mV throughout the 28 days period. These observations suggest the release of acidic products and formation of neutrally charged species during the reaction.

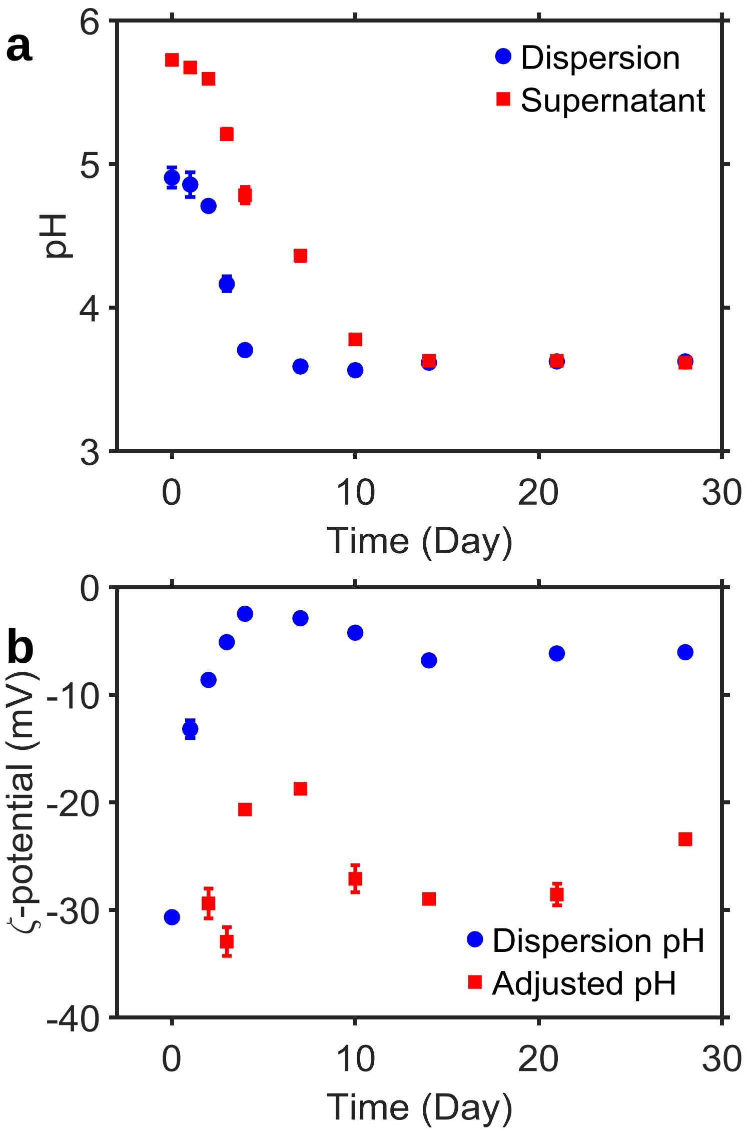

Figure S5. The effect of storage on *pH* and ζ-potential of dMXene dispersion. (a) *pH* of dMXene dispersions and supernatants during the storage period. (b) ζ-potential of original and neutralized dMXene dispersions during the storage period.

2.3. Preparation of stable dMXene samples

The preparation of various dMXene dispersions are summarized in **Figure 2** (main text). Using the solvent exchange (SE) approach, we prepared dMXene dispersions in eleven organic solvents from the dMXene dispersion (**Figure S6**). We also used the re-dispersion (RD) approach to obtain dMXene dispersions in seven organic solvents by processing the

mlMXene powder. We compared the above dMXene samples against the aqueous dMXene dispersion under continuous bubbling of argon (Ar-dMXene) for a period of 28 days. Our observations indicated that the SE route was more versatile than the RD route and could be used for a wide range of organic solvents. Most organic solvents (except toluene and hexane) worked for the SE route, while the RD approach only worked for ethanol, DMF, and DMSO. SEM observations of dMXene samples on day 0 revealed that the flakes retained their sheet-like morphology in all of the three processing approaches (**Figure S6**). The SE approach also allowed for the sequentially transfer of dMXene nanosheets into other solvents (**Figure S7**).

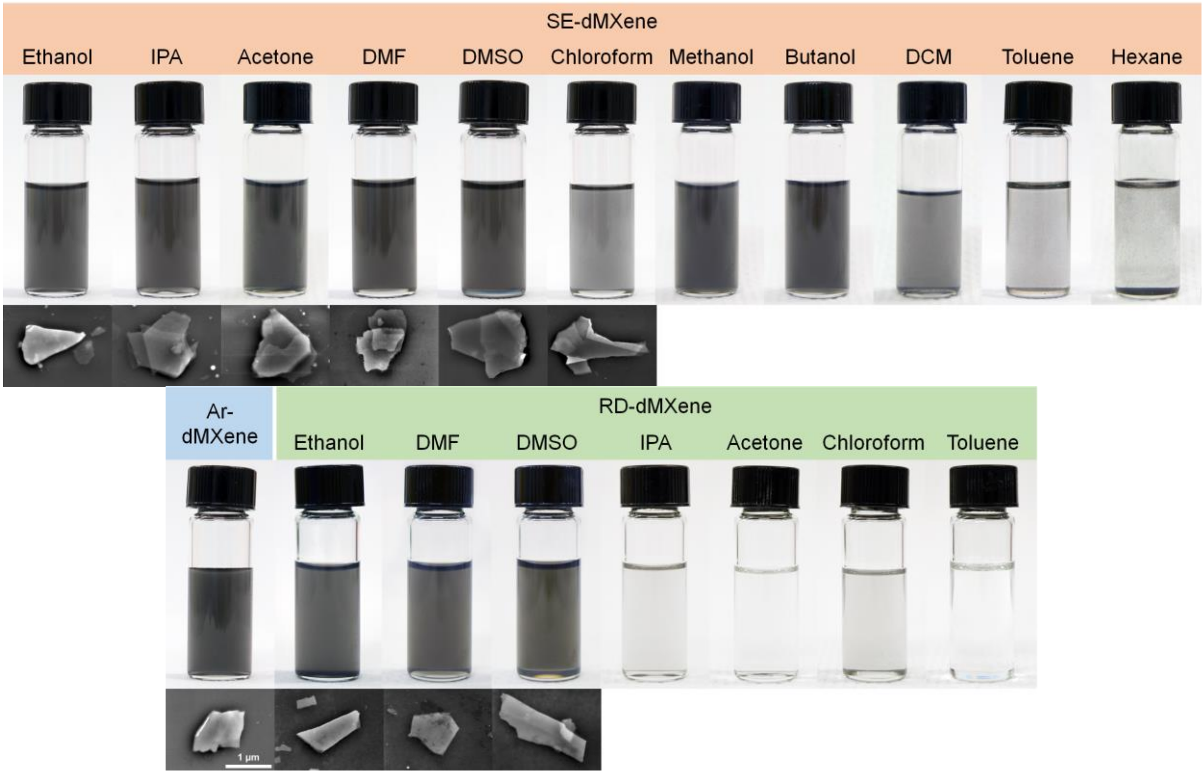

Figure S6. Digital photographs and SEM images of the Ar-, SE-, and RD-dMXene dispersions taken immediately after processing. All dMXene dispersions retained their sheet-like morphology after processing.

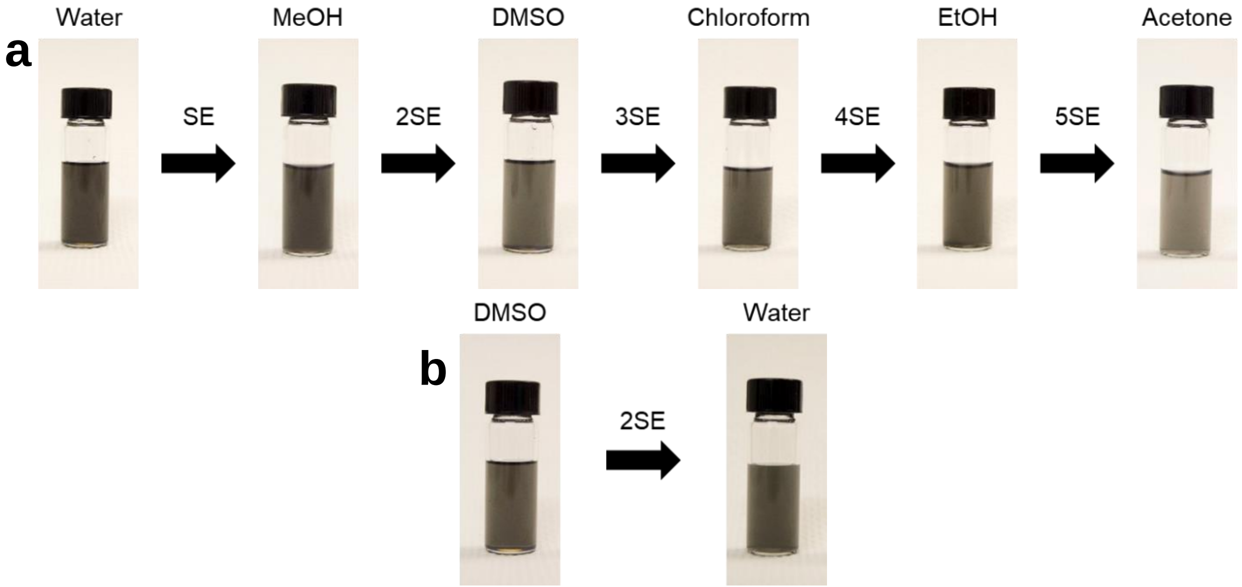

Figure S7. dMXene dispersions obtained by repeated solvent exchange (*n*SE) process. (a) Photographs of dMXene dispersions sequentially transferred to different solvents via repeated SE process. (b) Photographs showing the SE-dMXene dispersion in DMSO after storage for 28 days, which has been transferred back to water via another SE process.

2.4. Comparison of UV-Vis absorbance of dMXene samples

In contrast to aqueous dMXene dispersions, the UV-Vis absorbance spectra of the SE-dMXene and RD-dMXene dispersions remained almost the same with no detectable sign of oxidation or degradation of MXene (**Figure S8**).

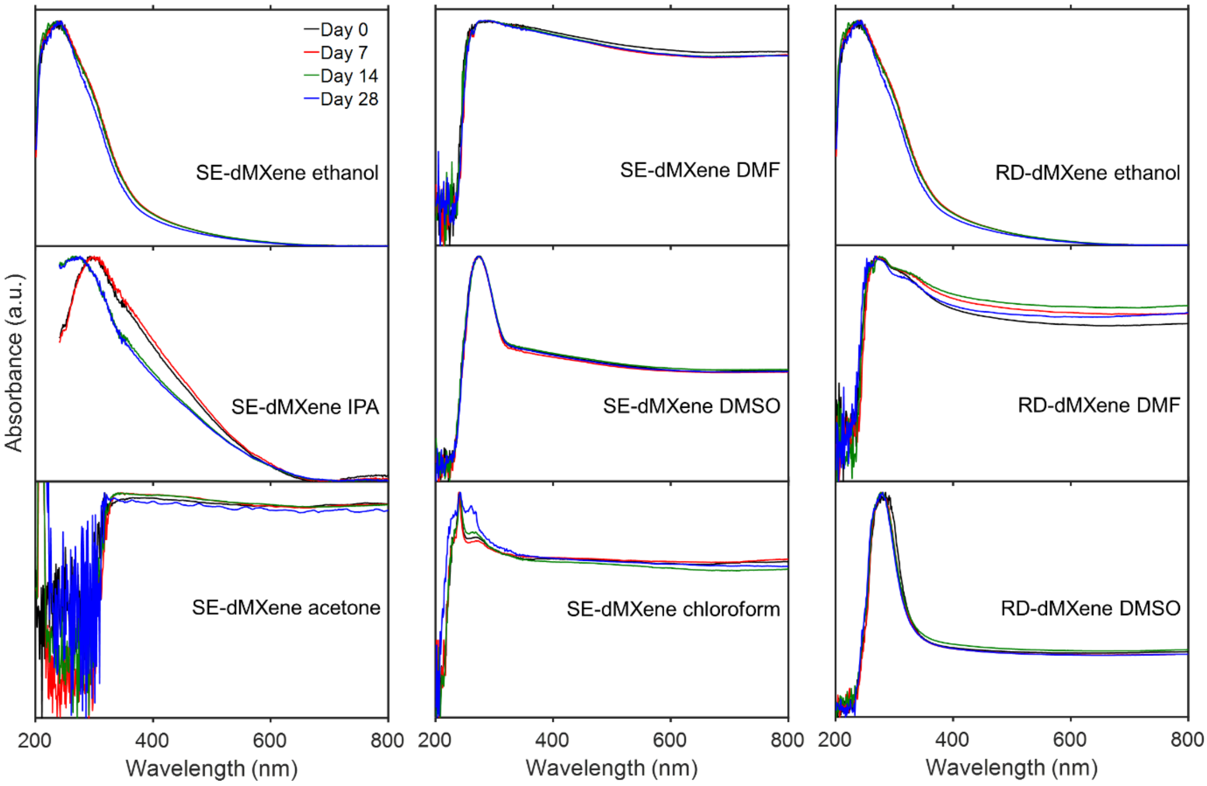

Figure S8. UV-Vis absorbance spectra for SE- and RD-dMXene dispersions over 28 days. The absorbance spectra of the dispersions remained almost the same in all samples.

2.5. Size measurements of dMXene samples

Dynamic light scattering measurements (**Figure S9**) showed an increase in the mean size from ~578 nm to ~2,610 nm for the dMXene dispersion stored in the ambient condition. Nevertheless, the increase in particle size was less obvious for the Ar-dMXene dispersion. Except for SE-dMXene in ethanol and IPA that showed slightly larger sizes in DLS measurements, all other dispersions on day 0 were of similar sizes to the original dMXene dispersion. Also, the sizes of SE-dMXenes and RD-dMXenes remained relatively unchanged over the storage period. Compared to the HF-derived $Ti_3C_2T_x$ MXene dispersions reported previously,[21] our dispersions contain MXene sheets with larger sizes. For instance, for dispersions in ethanol obtained by similar RD route, we measured a z-average of 2,864 nm, while a z-average in the range of 300-400 nm was observed for the HF-based MXene. This difference in particle size could be attributed to the differences in the synthesis approach.

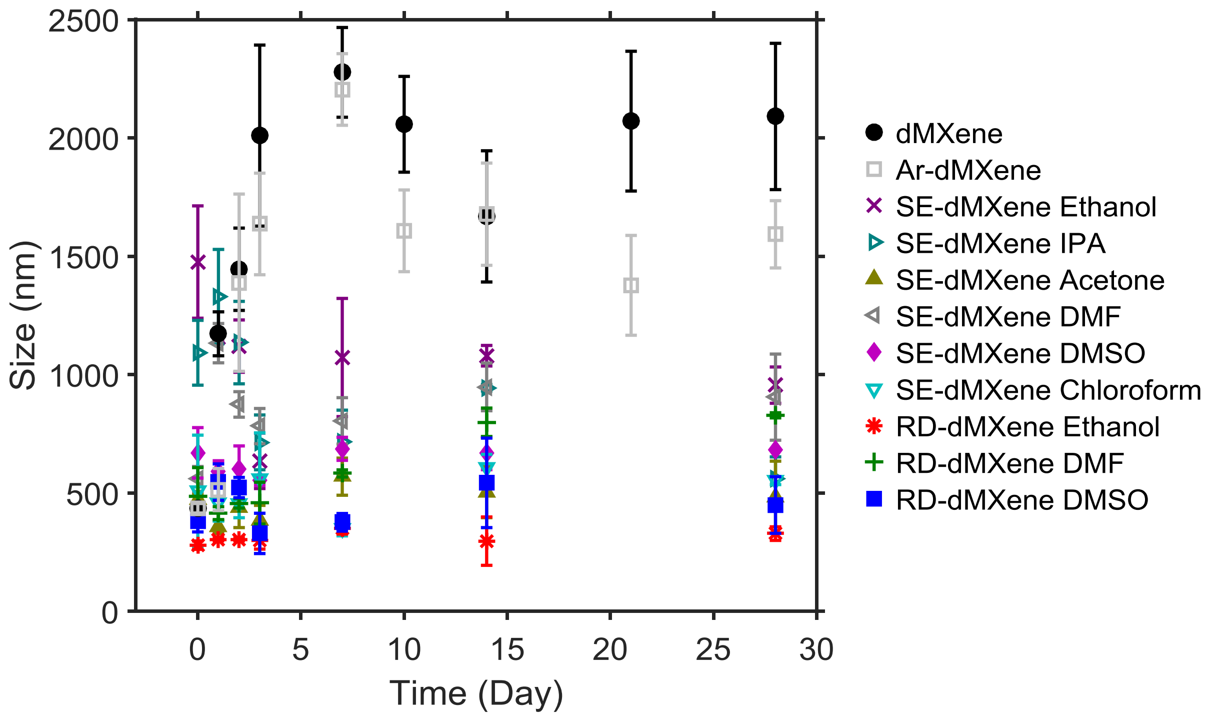

Figure S9. Size measurement for different dMXene dispersions over 28 days.

2.6. XRD, Raman spectroscopy and XPS analyses of dMXene samples

The characterization of powder samples collected from dMXene dispersions obtained by the SE, RE, and Ar approaches on day 0 showed signatures of delaminated MXene *i.e.* (002) diffractions in the XRD spectra (**Figure S10a**), and the A_{1g} mode of MXene and the D and G bands vibrations in the Raman spectra (**Figure S10b**). The shifts in the (002) diffraction to lower 2θ (**Figure S10a**) were observed to depend on the solvent used and the preparation of the dispersions. The highest downshift was observed for SE-dMXene in DMSO with 2θ ~5.1° corresponding to a *d*-spacing of 17.2 Å, which was higher than that of dMXene in water (13.9 Å). This downshift in (002) diffraction and the corresponding increase in the inter-layer spacing has been previously attributed to the intercalation of solvents between MXene layers.[2,3,22]

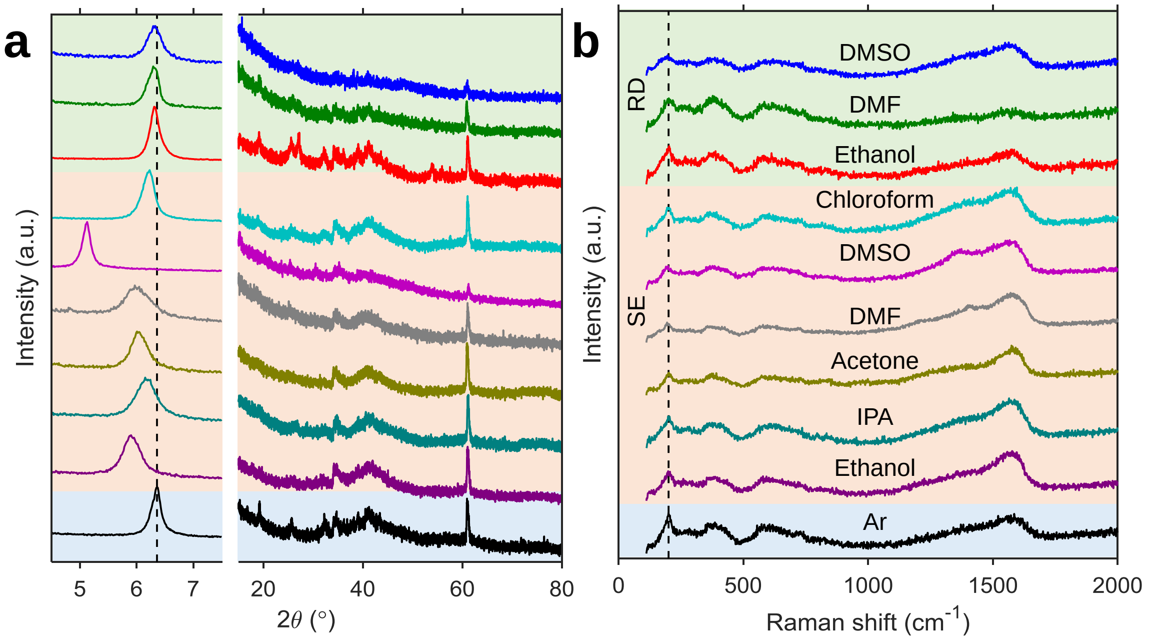

Figure S10. Characterizations of Ar-, SE-, and RD-dMXene dispersions on day 0. (a) XRD patterns of dMXene powders collected from different dispersions. The dashed line in (a) represents the (002) diffraction of the freshly prepared dMXene. (b) Raman spectra of the Ar-, SE-, and RD-dMXene powders collected from their dispersions. The color coding and positioning of the samples within the figure in (a) are the same as the samples in (b).

The analysis of XPS spectra for dMXene powder samples in Ti 2p region (**Figure S11a**) showed that over prolonged storage of the aqueous dispersion and as the result of oxidation, the Ti-C bond ratio decreased and Ti-O (IV) contribution increased whereby on day 28, ~99 % of the intensity came from the Ti-O (IV) component. The XPS observation hints at the formation of TiO_2 oxidation by-product. Also, in C 1s region of the XPS spectra (**Figure S11b**), gradual Ti-C bond destruction was also observed for the dMXene sample. The Ti-C bond was present in the XPS spectra of SE-dMXene in DMSO on day 28 in both Ti 2p and C 1s regions, indicating the absence of oxidation. **Table S1** and **Table S2** summarize the results of peak fitting on high-resolution XPS spectra in the Ti 2p and C 1s regions respectively for different samples.

In the previous report by Zhang *et al.*[10], the oxidation process of HF-etched $Ti_3C_2T_x$ MXene dispersion was studied. In this work, $Ti_3C_2T_x$ MXene was prepared using LiF/HCl as the etching agent. We observed slightly different oxidation process where the carbon content gradually decreased as the oxidation progressed. This is in contrast to the previous report where the carbon layer developed into disordered carbon upon MXene oxidation. A previous study found that the oxidation of MXene depends on the synthesis route.[23] In this study, we observed that the degradation of $Ti_3C_2T_x$ MXene into rutile TiO_2 occurs randomly on the sheet surface without noticeable preference to the edges. This indicates that the oxidation is likely to start from the exposed Ti- of MXene on the surface. The variation in synthesis methods could introduce different amount of defect on the $Ti_3C_2T_x$ MXene, which could result in a different oxidation behavior due to the differences in the exposed Ti-. However, further experiments and modelling are required to clearly elucidate the mechanism and kinetics of MXene oxidation.

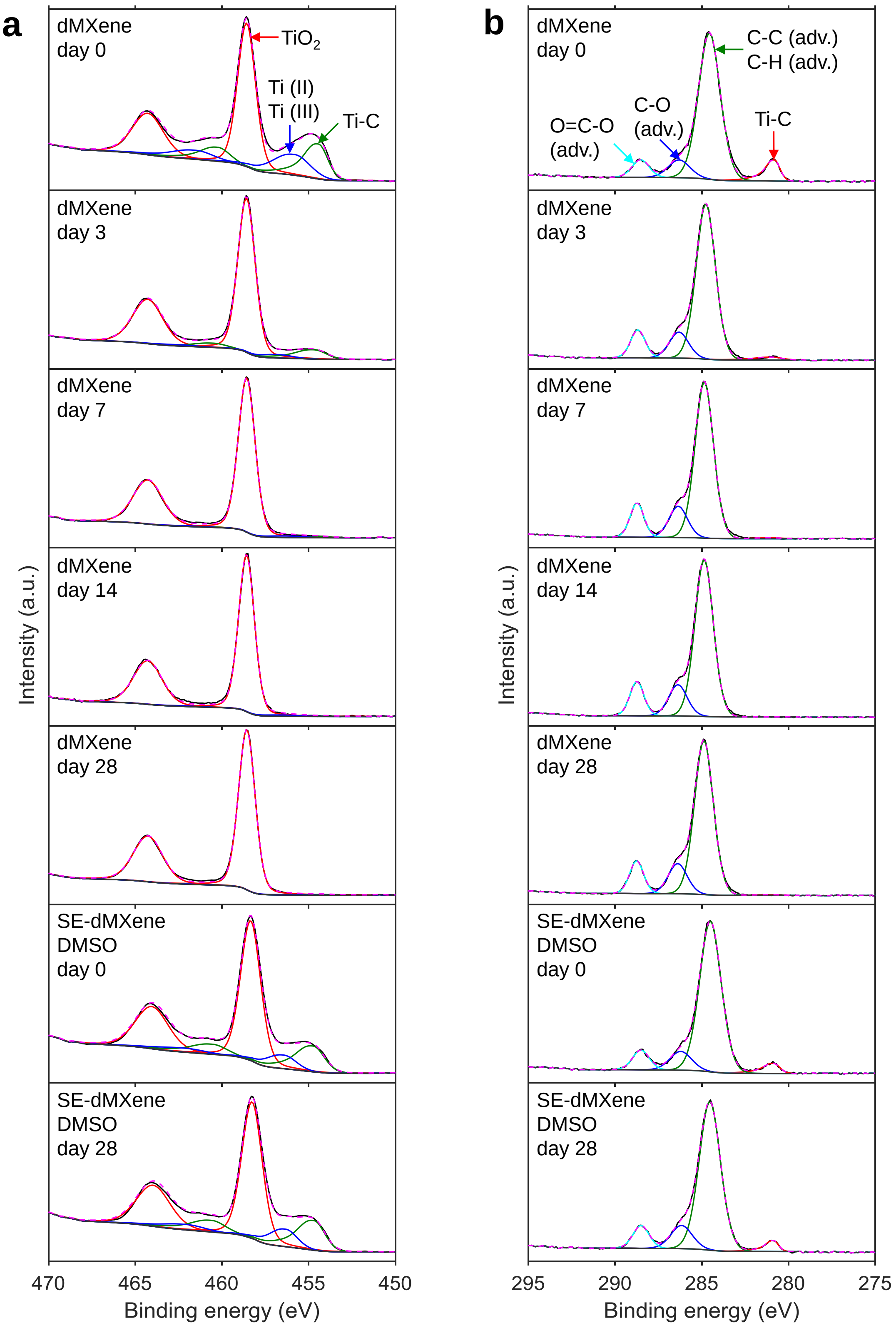

Figure S11. High-resolution XPS spectra of dMXene and SE-dMXene in DMSO powder samples at different time periods. XPS spectra for (a) Ti 2p and (b) C 1s regions.

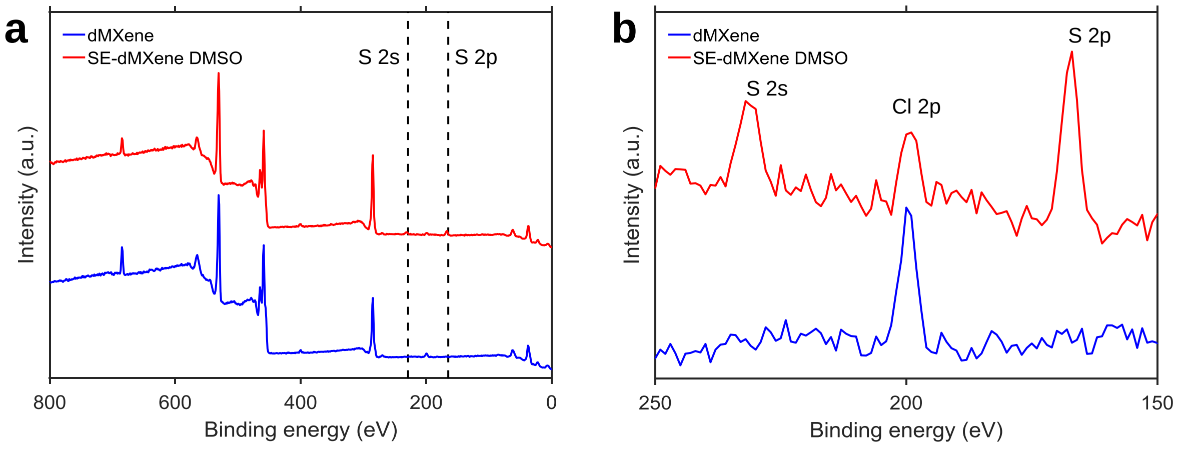

Figure S12. Comparison of XPS survey spectra of SE-dMXene in DMSO with dMXene (water). (a) The entire binding energy range and (b) between 150-250 eV showing S 2s and S 2p peaks related to the intercalation of DMSO solvent.

Table S1. Peak fitting results of high-resolution XPS spectra in the Ti 2p region for MAX phase and as-synthesized and stored dMXene samples in water and SE-dMXene in DMSO. In columns 2 and 3, the numbers out of brackets are peak positions in terms of binding energy (BE) and full width at half maximum (FWHM) of the peaks for Ti $2p_{3/2}$ respectively. The numbers in brackets show BE peak positions and FWHMs for Ti $2p_{1/2}$

	BE [eV]	FWHM [eV]	Assignment	%
MAX	453.5 (459.5)	0.9 (0.9)	Ti-Al	7.3
Ti $2p_{3/2}$ ($2p_{1/2}$)	454.2 (460.0)	1.3 (1.4)	Ti-C	17.8
	455.5 (461.2)	1.8 (2.3)	Ti(II)/Ti(III)	16.0
	458.2 (463.9)	1.5 (2.2)	Ti(IV) oxides	58.9
dMXene Day 0	454.4 (460.2)	1.3 (1.6)	Ti-C	20.7
Ti $2p_{3/2}$ ($2p_{1/2}$)	455.8 (461.5)	2.1 (2.4)	Ti(II)/Ti(III)	18.2
	458.6 (464.3)	1.2 (2.1)	Ti(IV) oxides	61.1
dMXene Day 3	454.7 (460.5)	1.7 (1.8)	Ti-C	9.9
Ti $2p_{3/2}$ ($2p_{1/2}$)	456.8 (462.5)	1.9 (2.5)	Ti(II)/Ti(III)	3.7
	458.6 (464.3)	1.2 (2.0)	Ti(IV) oxides	86.4
dMXene Day 7	454.3 (460.1)	1.5 (1.6)	Ti-C	1.1
Ti $2p_{3/2}$ ($2p_{1/2}$)	456.3 (462.0)	2.2 (2.3)	Ti(II)/Ti(III)	2.3
	458.6 (464.3)	1.2 (2.0)	Ti(IV) oxides	96.6
dMXene Day 14	454.2 (460.0)	1.5 (1.6)	Ti-C	0.5
Ti $2p_{3/2}$ ($2p_{1/2}$)	456.5 (462.2)	2.2 (2.3)	Ti(II)/Ti(III)	2.0
	458.6 (464.3)	1.1 (1.9)	Ti(IV) oxides	97.5
dMXene Day 28	454.2 (460.0)	1.5 (1.6)	Ti-C	0.5
Ti $2p_{3/2}$ ($2p_{1/2}$)	456.4 (462.1)	2.2 (2.3)	Ti(II)/Ti(III)	0.7
	458.6 (464.3)	1.1 (2.0)	Ti(IV) oxides	98.7
SE-dMXene DMSO Day 0	454.7 (460.5)	1.6 (2.0)	Ti-C	19.6
Ti $2p_{3/2}$ ($2p_{1/2}$)	456.4 (462.1)	1.7 (2.8)	Ti(II)/Ti(III)	10.2
	458.4 (464.1)	1.4 (2.3)	Ti(IV) oxides	70.2
SE-dMXene DMSO Day 28	454.7 (460.6)	1.5 (1.9)	Ti-C	20.8
Ti $2p_{3/2}$ ($2p_{1/2}$)	456.3 (462.0)	1.6 (2.5)	Ti(II)/Ti(III)	11.8
	458.3 (464.0)	1.4 (2.3)	Ti(IV) oxides	67.4

Table S2. Peak fitting results of high-resholution XPS spectra in the C 1s region for MAX phase and as-synthesized and stored dMXene samples in water and SE-dMXene in DMSO. Hydrocarbon and oxyhydrocarbon species arise mostly from the tape used to immobilize the particles during the analysis, or from adventitious (adv.) carbon contamination

	BE [eV]	FWHM (eV)	Assignment	%
MAX	280.9	0.7	Ti-C	8.5
C 1s	284.6	1.4	C--C/C--H (adv.)	44.3
	285.7	1.9	C--O (adv.)	37.1
	288.6	1.9	O=C--O (adv.)	10.1
dMXene Day 0	280.8	0.8	Ti-C	4.3
C 1s	284.7	1.4	C--C/C--H (adv.)	73.4
	286.3	1.4	C--O (adv.)	12.0
	288.6	1.0	O=C--O (adv.)	10.2
dMXene Day 3	281.0	1.5	Ti-C	2.4
C 1s	284.8	1.3	C--C/C--H (adv.)	74.7
	286.3	1.3	C--O (adv.)	12.5
	288.7	1.0	O=C--O (adv.)	10.3
dMXene Day 7	281.0	1.5	Ti-C	0.8
C 1s	284.9	1.3	C--C/C--H (adv.)	72.7
	286.4	1.3	C--O (adv.)	14.5
	288.7	0.9	O=C--O (adv.)	12.0
dMXene Day 14	281.0	1.5	Ti-C	0.0
C 1s	284.9	1.3	C--C/C--H (adv.)	73.2
	286.4	1.3	C--O (adv.)	14.5
	288.7	1.0	O=C--O (adv.)	12.2
dMXene Day 28	281.0	1.5	Ti-C	0.0
C 1s	284.9	1.3	C--C/C--H (adv.)	73.4
	286.4	1.3	C--O (adv.)	16.6
	288.8	1.0	O=C--O (adv.)	12.0
SE-dMXene DMSO Day 0	280.9	0.9	Ti-C	4.8
C 1s	284.5	1.5	C--C/C--H (adv.)	77.7
	286.2	1.5	C--O (adv.)	9.7
	288.5	1.1	O=C--O (adv.)	7.9
SE-dMXene DMSO Day 28	280.9	0.7	Ti-C	4.1
C 1s	284.5	1.5	C--C/C--H (adv.)	75.3
	286.2	1.5	C--O (adv.)	11.8
	288.5	1.1	O=C--O (adv.)	8.8

Table S3. Comparison of the mechanical and electrical properties of pure PCL, SE-dMXene/PCL and RD-dMXene/PCL fibers

Fiber	Diameter [μm]	Tensile Strength [MPa]	Strain at break [%]	Conductivity [mS cm^{-1}]
Pure PCL	117 ± 1.22	7.03 ± 1.69	1012 ± 110	N/A
SE-dMXene/PCL	127 ± 0.84	4.15 ± 0.39	770 ± 52.6	1.84 ± 0.12
RD-dMXene/PCL	109 ± 1.30	5.20 ± 0.87	622 ± 104	N/A

Supplementary references

[1] M. Ghidiu, M. R. Lukatskaya, M.-Q. Zhao, Y. Gogotsi, M. W. Barsoum, *Nature* **2014**, *516*, 78.

[2] M. R. Lukatskaya, O. Mashtalir, C. E. Ren, Y. Dall'Agnese, P. Rozier, P. L. Taberna, M. Naguib, P. Simon, M. W. Barsoum, Y. Gogotsi, *Science* **2013**, *341*, 1502.

[3] O. Mashtalir, M. Naguib, V. N. Mochalin, Y. Dall'Agnese, M. Heon, M. W. Barsoum, Y. Gogotsi, *Nat. Commun.* **2013**, *4*, 1716.

[4] M. Naguib, V. N. Mochalin, M. W. Barsoum, Y. Gogotsi, *Adv. Mater.* **2014**, *26*, 992.

[5] B. Anasori, M. R. Lukatskaya, Y. Gogotsi, *Nat. Rev. Mater.* **2017**, *2*, 16098.

[6] H. Ghassemi, W. Harlow, O. Mashtalir, M. Beidaghi, M. R. Lukatskaya, Y. Gogotsi, M. L. Taheri, *J. Mater. Chem. A* **2014**, *2*, 14339.

[7] M. Naguib, O. Mashtalir, M. R. Lukatskaya, B. Dyatkin, C. Zhang, V. Presser, Y. Gogotsi, M. W. Barsoum, *Chem. Commun.* **2014**, *50*, 7420.

[8] C. Peng, X. Yang, Y. Li, H. Yu, H. Wang, F. Peng, *ACS Appl. Mater. Interfaces* **2016**, *8*, 6051.

[9] G. Zou, J. Guo, Q. Peng, A. Zhou, Q. Zhang, B. Liu, *J. Mater. Chem. A* **2016**, *4*, 489.

[10] C. Zhang, S. Pinilla, N. McEvoy, C. P. Cullen, B. Anasori, E. Long, S.-H. Park, A. Seral-Ascaso, A. Shmeliov, D. Krishnan, C. Morant, X. Liu, G. S. Duesberg, Y.

Gogotsi, V. Nicolosi, *Chem. Mater.* **2017**, *29*, 4848.

[11] O. Mashtalir, K. M. Cook, V. N. Mochalin, M. Crowe, M. W. Barsoum, Y. Gogotsi, *J. Mater. Chem. A* **2014**, *2*, 14334.

[12] T. Hu, J. Wang, H. Zhang, Z. Li, M. Hu, X. Wang, *Phys. Chem. Chem. Phys.* **2015**, *17*, 9997.

[13] O. Frank, M. Zukalova, B. Laskova, J. Kürti, J. Koltai, L. Kavan, *Phys. Chem. Chem. Phys.* **2012**, *14*, 14567.

[14] J. Yan, G. Wu, N. Guan, L. Li, Z. Li, X. Cao, *Phys. Chem. Chem. Phys.* **2013**, *15*, 10978.

[15] M. Ashton, K. Mathew, R. G. Hennig, S. B. Sinnott, *J. Phys. Chem. C* **2016**, *120*, 3550.

[16] L.-Y. Gan, D. Huang, U. Schwingenschlögl, *J. Mater. Chem. A* **2013**, *1*, 13672.

[17] R. F. Voitovich, É. A. Pugach, *Powder Metall. Met. Ceram.* **1972**, *11*, 132.

[18] Y. Qin, W. Lu, D. Zhang, J. Qin, B. Ji, *Mater. Sci. Eng. A* **2005**, *404*, 42.

[19] Y. Ying, Y. Liu, X. Wang, Y. Mao, W. Cao, P. Hu, X. Peng, *ACS Appl. Mater. Interfaces* **2015**, *7*, 1795.

[20] M. Alhabeb, K. Maleski, T. S. Mathis, A. Sarycheva, C. B. Hatter, S. Uzun, A. Levitt, Y. Gogotsi, *Angew. Chemie Int. Ed.* **2018**, *57*, 5444.

[21] K. Maleski, V. N. Mochalin, Y. Gogotsi, *Chem. Mater.* **2017**, *29*, 1632.

[22] M. Naguib, Y. Gogotsi, *Acc. Chem. Res.* **2015**, *48*, 128.

[23] A. Lipatov, M. Alhabeb, M. R. Lukatskaya, A. Boson, Y. Gogotsi, A. Sinitskii, *Adv. Electron. Mater.* **2016**, *2*, 1600255.
